# One‐step Centimeter‐Scale Growth of Sub‐100‐nm Perovskite Single‐Crystal Arrays in Ambient Air for Color Painting

**DOI:** 10.1002/advs.202415105

**Published:** 2025-02-03

**Authors:** Guannan Zhang, Zhao Sun, Zhuofei Gan, Chuwei Liang, Liyang Chen, Hongbo Mo, Yuanzhi Jiang, Mingjian Yuan, Aleksandra B. Djurišić, Ji Tae Kim, Wen‐Di Li

**Affiliations:** ^1^ Department of Mechanical Engineering The University of Hong Kong Hong Kong SAR 999077 P. R. China; ^2^ Department of Physics The University of Hong Kong Hong Kong SAR 999077 P. R. China; ^3^ State Key Laboratory of Advanced Chemical Power Sources Key Laboratory of Advanced Energy Materials Chemistry (Ministry of Education) Frontiers Science Center for New Organic Matter College of Chemistry Nankai University Tianjin 300073 P. R. China; ^4^ Department of Mechanical Engineering Korea Advanced Institute of Science and Technology (KAIST) Science Town Daejeon 34141 Republic of Korea

**Keywords:** ambient environment, full‐color, perovskite, single crystal, sub‐100 nm

## Abstract

Halide perovskite single crystals have demonstrated enormous potential for next‐generation integrated optoelectronic devices. However, there is a lack of a facile method to realize the controllable growth of large‐scale, high‐quality, and high‐resolution perovskite single crystal arrays on diverse types of substrates, which hinders their application in practical scenarios. Here, a one‐step wettability‐guided blade coating approach is reported for the rapid in situ crystallization of large‐scale, multicolor, and sub‐100 nm perovskite single‐crystal arrays in the ambient environment. By this strategy, the physical dimensions of perovskite single crystals can be precisely regulated from 90 to 260 nm, with a size variation coefficient < 10% and an area of over 900 mm^2^. All three typicalhalogen perovskites for multi‐color luminescence, CsPbX_3_ (X = Cl, Br, I) and their mixtures (Cl/Br or Br/I systems), are appliable to this fabrication process through the demonstration of complex RGB patterns with remarkable photoluminescence properties. Moreover, various rigid substrates such as silicon oxide (SiO_2_), silicon (Si), and glass can also be used to construct the wettability‐constrast templates where perovskite crystal nucleate and grow. After that, the perovskite single‐crystal arrays or complex patterns can be transferred onto flexible substrates, for instance, COC. This method combines convenient solution processing with conventional photolithography to prepare the high‐resolution, large‐area, and superior‐quality perovskite single crystal arrays in a high‐throughput manner, showing great potential in the integration of perovskite nano‐optoelectronic devices and chips.

## Graphical abstract

Researchers have developed a one‐step wettability‐guided blade coating approach for the rapid in situ crystallization of large‐scale, multicolor, and sub‐100 nm perovskite single‐crystal arrays in the ambient environment. This strategy can be widely extended to various types of halide perovskites and substrates, showing great potential in the integration of perovskite nano‐optoelectronic devices and chips.

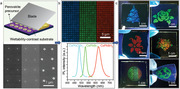

## 1 Introduction

Halide perovskite single crystals process remarkable optoelectronic characteristics, such as high carrier mobility,^[^ ^1^, ^2^, ^3^ ^]^ long diffusion length,^[^ ^4^, ^5^, ^6^ ^]^ excellent crystallinity,^[^ ^7^, ^8^ ^]^ low trap state density,^[^ ^9^, ^10^, ^11^ ^]^ which have been widely used in diverse optoelectronic devices including PeLEDs,^[^ ^12^, ^13^, ^14^, ^15^ ^]^ lasers,^[^ ^16^, ^17^, ^18^, ^19^ ^]^ photodetectors,^[^ ^20^, ^21^, ^22^, ^23^ ^]^ and solar cells.^[^ ^24^, ^25^ ^]^ Some recent studies have shown that micro/nano‐perovskite single crystals of specific sizes and dimensions process more superior advantages over bulk crystals.^[^ ^26^, ^27^, ^28^ ^]^ For example, perovskite quantum dots present high photoluminescence quantum yield (PLQY) and tunable emission spectrum due to the unique quantum confinement effect.^[^ ^29^, ^30^ ^]^ Besides, perovskite nano‐single crystals can constitute tiny optical resonant cavity, which can provide high‐speed channels for the directional transmission and propagation of photons and charge carriers, thereby bringing greater gain to low‐threshold laser applications.^[^ ^31^, ^32^, ^33^, ^34^ ^]^ In addition, perovskite single‐crystal micro/nano‐plates meet the thickness requirements of optoelectronic devices. And the structures with low defect and without grain boundary characteristics facilitate the separation of charge carriers, which are of great significance to improve the performance of devices.^[^ ^35^ ^]^ For above advantages, growing perovskite single crystals at the nanoscale provides a potential method to achieve on‐chip integrated optoelectronic devices. Therefore, patterning these perovskite single crystals on various substrates at the nanoscale with high resolution and precise positioning accuracy plays a key role in the integration of functional nano‐devices. Due to the incompatibility of perovskites with polar solvents, the commonly used photolithography and etching techniques are not suitable for fabricating patterned perovskite single crystal arrays.^[^ ^33^ ^]^

With this regard, researchers have demonstrated two mainstream methods, maskless and mask‐based patterning methods. Typical maskless patterning methods including printing^[^ ^36^, ^37^, ^38^ ^]^ and particle beam‐/laser‐assisted patterning technologies,^[^ ^39^, ^40^, ^41^ ^]^ provide high controllability. Inkjet printing^[^ ^42^, ^43^ ^]^ and electrohydrodynamic (EHD) printing^[^ ^44^, ^45^ ^]^ have been widely used for fast, scalable, and mask‐free patterning of perovskite. Nevertheless, printing methods are normally challenging to achieve a resolution and placement accuracy at the sub‐micron level. To further improve the resolution, various particle beam‐/laser‐assisted patterning methods including focused ion beam (FIB) milling,^[^ ^46^ ^]^ electron‐beam lithography (EBL),^[^ ^47^, ^48^ ^]^ and laser machining^[^ ^39^, ^49^ ^]^ have also been used for perovskite patterning. Those methods can directly remove selected regions and write patterns on demand with resolutions up to tens of nanometers. However, the perovskite is easily damaged from beam source and ultraviolet light source, and the fabrication efficiency is always a great challenge.

In terms of mask‐based patterning methods, a prepatterned template is usually prepared to further selectively grow perovskite structure, which exhibits excellent scalability. On one hand, prepatterned physical templates are fabricated to guide the growth of perovskite.^[^ ^50^, ^51^, ^52^, ^53^ ^]^ For instance, as a high‐throughput and scalable patterning method, nanoimprint lithography (NIL) is widely used to prepare high‐precision templates with features below tens of nanometers in size.^[^ ^54^ ^]^ These physical templates are then employed as spatial confinements to guide the growth of perovskite structures. Inevitably, however, such templates are usually expensive and complicated to prepare, and it usually needs several hours to realize the slow growth of perovskite single crystals. On the other hand, chemically functionalized templates provide a remarkable chance to generate patterned perovskite nano‐structures.^[^ ^27^, ^33^, ^55^, ^56^ ^]^ By preparing a substrate with a wettability contrast pattern, perovskite materials can be selectively grown to form a pattern. This method can reduce manufacturing costs, but it is still difficult to achieve sub‐100 nanometer structures. To date, these methods mentioned above have yielded polycrystalline and single‐crystalline perovskite structures with feature sizes ranging from sub‐micron to tens or microns, yet no method has been demonstrated for synthesizing large‐area, sub‐100 nm perovskite single crystal arrays with high spatial resolution and positioning accuracy on arbitrary substrates in a high‐throughput manner.^[^ ^41^ ^]^

Herein, we propose a one‐step solution processing approach to realize centimeter‐scale growth of sub‐100 nm perovskite single‐crystal arrays in ambient environment. Interference lithography technology is introduced to prepare photoresist nanopillar array on the substrate, and then a surface treatment using trichloro(1H,1H,2H,2H‐perfluorooctyl)silane (FDTS) creates a wettability contrast pattern on the substrate surface with the photoresist mask. After that, perovskite precursor solution is selectively blade‐coated on the wettability‐patterned substrate. This strategy enables centimeter‐level controlled growth of single‐crystal inorganic halide perovskite arrays with precisely controllable crystal size (from 90 to 260 nm), and is compatible with various rigid and flexible substrates (SiO_2_, Si, glass, COC). Notably, this method is applicable to all three types of halogen perovskites CsPbX_3_ (X = Cl, Br, I) and their mixtures to cover a broad color range. Complex RGB patterns are successfully generated on diverse substrates, exhibiting great uniformity and outstanding photoluminescence (PL) properties. Therefore, this method combines relatively mature photolithography and low‐cost solution processing to prepare large‐area, high‐quality perovskite single crystal arrays in a high throughput manner under ambient conditions, paving the way for integrated chips and functional optoelectronic devices.

## 2 Results and Discussion

**Figure 1:**
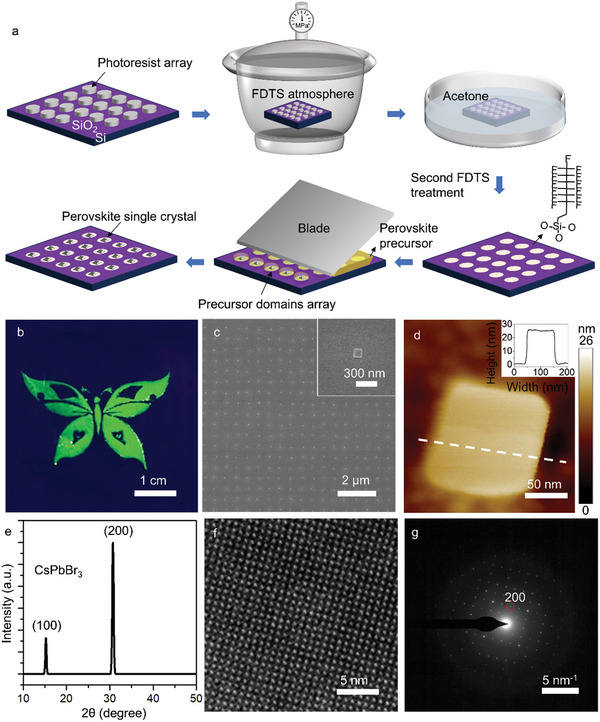
a) Schematic illustration of the method to prepare perovskite single crystal arrays. Periodically patterned substrate is modified by FDTS monolayer, and lyophilic/lyophobic substrate with appropriate surface wettability difference is constructed through twice fluorination treatments. The perovskite precursor is then confined to relatively lyophilic regions via a one‐step blade coating approach. As the solution evaporates, the perovskite crystals nucleate and grow, eventually forming perovskite single crystal arrays. b) Fluorescence micrograph of CsPbBr_3_ complex pattern fabricated by the one‐step blade coating strategy. c) SEM image of a perovskite single crystal array with a period of 1 µm. Inset illustrates the enlarged view of an individual CsPbBr_3_ crystal. d) AFM image of a single CsPbBr_3_ plate, and the insert is the height profile. e) XRD pattern of as‐synthesized 3^*^3 cm^2^ CsPbBr_3_ array. f) HRTEM image and g) SAED patterns of a single CsPbBr_3_ crystal.

**Figure** **1**a demonstrates the schematic flowchart of the one‐step solution preparation of perovskite single crystal arrays. Firstly, large‐scale photoresist arrays with different feature sizes are prepared by interference lithography. Figures S1 and S2 (Supporting Information) show the physical and schematic diagrams of interference lithography equipment, respectively. The principle of interference lithography is briefly explained in Note S1 (Supporting Information). Large‐scale 2D photoresist patterns can be obtained with high quality using the interference lithography equipment, as shown in Figure S3 (Supporting Information). Then the substrate with photoresist patterns on the surface is treated in a vapor phase FDTS treatment chamber, during which FDTS molecules react with the hydroxyl groups on the exposed substrate surface to functionalize the surface with fluorocarbon groups while photoresist‐covered area remains untreated, thereby the substrate surface can be patterned with wettability contrast after dissolving the photoresist. Here we further optimize this method by performing a second surface functionalization treatment on the substrate so that the lyophilic regions become slightly lyophobic to tailor the wettability contrast between the two regions. This surface treatment method processes strong universality, which is suitable for a variety of substrates such as silicon dioxide, silicon, and glass. After surface treatment, the perovskite precursor is applied through a blade coating process with a constant coating speed. The blade‐coating system consists of a blade, hot plate, and control system as well as other auxiliary equipment, as shown in Figure S4 and Note S2 (Supporting Information). Though pinning the three‐phase contact lines (TCL) at the edge of the lyophilic areas, the solution can be isolated and confined into the lyophilic region which was originally covered by photoresist in the first FDTS treatment process. As the contact line slips and the solvent evaporates, perovskite crystals nucleate and grow, eventually forming arrayed perovskite single crystal arrays.

By combining the traditional lithography technology with facile solution‐based process, this strategy is effective to prepare large‐area perovskite single crystal arrays. To demonstrate the scalability of this method, a pre‐patterned substrate with a lyophilic array of circular regions of 500 nm diameter is utilized to generate the perovskite array. Figure 1b depicts a CsPbBr_3_ ‘butterfly’ pattern consisting of over 100 000 000 perovskite pixels on a 3 × 3 cm^2^ silicon dioxide substrate, emitting uniform and bright green fluorescence. Figure S5 (Supporting Information) shows the physical pictures of ‘butterfly’ photoresist pattern and the sample after blade coating. As shown in Figure 1c, the scanning electron microscopy (SEM) illustrates the detailed morphology of the complex pattern, illustrating that the perovskite crystals are well aligned on the pre‐patterned substrate in a homogeneous rectangular shape with a crystal size (measured in diagonal dimension) of 150 nm. The pitch of the perovskite array is 1 µm, as determined by the photoresist pattern through the interference lithography patterning, showing good positioning accuracy. A larger area of perovskite single crystal array in the butterfly pattern is shown in Figure S6 (Supporting Information). Figure 1d illustrates the atomic force microscopy (AFM) image of CsPbBr_3_ crystal, showing that the thickness of perovskite crystal with 150‐nm diagonal size is around 26 nm.

To prove the single‐crystal nature of perovskite crystals, X‐ray diffraction (XRD) and transmission electron microscopy (TEM) were applied to study the crystal structure. The XRD results of large‐area ‘butterfly’ pattern are summarized in Figure 1e, presenting three strong diffraction peaks at 15.1 ° and 30.7 °, which are attributed to the (100), and (200) planes, respectively. Since the arrangement directions of the perovskite crystals are not completely consistent within the centimeter‐level XRD spot range, we further prove our hypothesis that the perovskite crystals are single crystals through TEM images. As shown in Figures 1f and S7 (Supporting Information), a set of (200) plane with a distance of 0.41 nm is presented in the high‐resolution TEM (HRTEM) image. The selected area electron diffraction (SAED) pattern shows a single set of sharp and symmetric diffraction spots that can be indexed to the orthorhombic CsPbBr_3_ structure (Figure 1g).

**Figure 2:**
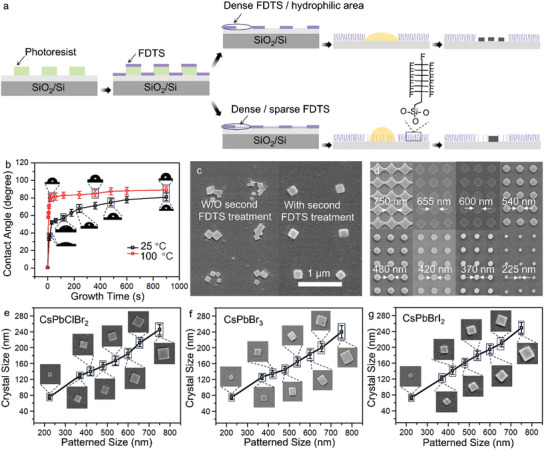
a) Schematic diagram of the interaction between droplet and surface under different wettability conditions. (Top row: wetting substrate, bottom row: nonwetting substrate. b) The relationship between contact angle and FDTS functionalization temperature as well as time. c) SEM image of crystallizations on the substrates with and without the second FDTS treatment. d) Periodic photoresist patterns with different feature sizes. e‐g) Dependence of crystal size of CsPbClBr_2_, CsPbBr_3_, CsPbBrI_2_ to pattern size, respectively.

Well‐controlled substrate conditions are the prerequisite for ensuring the quality of perovskite crystals. After dewetting, nucleation and growth, perovskite crystals can eventually form on substrates. The dewetting process mainly involves droplet confinement, solvent evaporation, and the evolution of the TCL. As shown in **Figure** **2**a, with the evaporation of solvent, the TCL is fixed on the edges of wetting regions (top row) but recedes on the surface with a secondary non‐wetting treatment (bottom row). More details about the solvent evaporation process are provided in Note S3 (Supporting Information). Previous research has also shown that the meniscus length on the lyophobic substrate is shorter than that of the untreated substrate,^[^ ^27^ ^]^ thus the solution has a longer meniscus in the lyophilic region to provide proper time for crystallization during the blade coating process. When the solution concentration reaches the critical supersaturation point, CsPbBr_3_ begins to nucleate on the surface. Regarding the wetting substrate, nucleation at multiple sites within one lyophilic area usually take place owing to the anchored TCL, thereby forming polycrystalline perovskite or multiple perovskite particles. Inversely, for properly treated non‐wetting substrates, the contact line can continuously slip and recede, which is more prone to the formation of single crystals. In order to obtain a suitable wettability contrast to ensure the ideal TCL behavior and formation of single crystals, the effects of fluorination temperature and time on the wettability (contact angle) of the substrate are studied, as shown in Figure 2b, Tables S1 and S2 (Supporting Information). According to these experimental results, the patterned substrate is heated at 100 °C in a FDTS atmosphere for 15 min, by which the contact angle of the region not covered by the photoresist increases to 89 °. After washing the photoresist patterns, the above substrate is subjected to a second FDTS treatment at 25 °C for 2 min so that the contact angle of the area covered by the photoresist increases from 0.8 ° to 57 °, while the contact angle of other regions remains constant. Figure 2c shows the effect of second FDTS treatment on perovskite crystallization, which is beneficial to the formation of perovskite single crystals rather than polycrystals.

Other than crystal structure, precise control of crystal size at the nanoscale is essential to integrated nanodevices. Hence, the relationship between pattern size and crystal morphology is studied. By controlling the exposure dose, the size of the photoresist pattern can be accurately controlled at the nanometer scale (Figure 2d). Crystal size is mainly determined by the precursor volume occupied by the relatively lyophilic region at a certain concentration. Thus, the crystal size can be tuned by changing the diameter of the photoresist pattern. Figure 2e–g illustrates the linear relationship between the perovskite crystal size and the pattern size of CsPbClBr_2_, CsPbBr_3_, and CsPbBrI_2_, respectively, demonstrating reproducible and precise preparation of perovskite single crystals ranging from tens to hundreds of nanometers. When the diameter of the photoresist array changes from 225 to 750 nm, the average size of the crystals (diagonal length) varies from 75 to 240 nm. Moreover, the size of crystals distributes in a narrow range, demonstrating excellent uniformity and consistency of as prepared single‐crystal arrays. The single crystal properties of CsPbClBr_2_ and CsPbBrI_2_ are confirmed in Figures S8 and S9 (Supporting Information).

**Figure 3:**
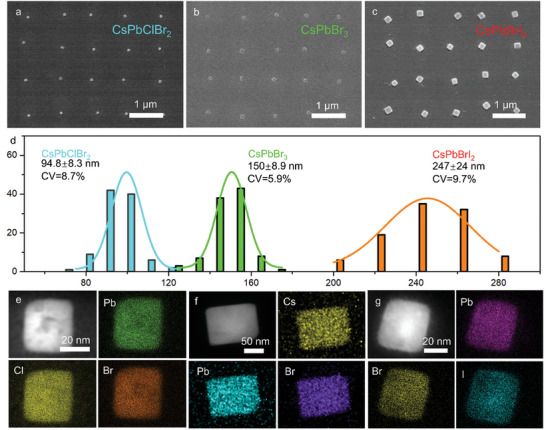
SEM images of a) CsPbClBr_2_, b) CsPbBr_3,_ and c) CsPbBrI_2_ single crystal arrays with different sizes. b) Histogram of perovskite nanoplate sizes on the above three samples. EDS mappings of e) CsPbClBr_2_, f) CsPbBr_3,_ and g) CsPbBrI_2_ single crystals.

Based on the above study, CsPbBr_3_ single crystal arrays are realized by one‐step growth method, which can also be extended to other halogen perovskites. **Figure** **3**a–c depicts SEM images of three types of perovskite single crystal arrays, showing regular outlines and ordered arrangement. The volume of the precursor domains in relatively hydrophilic area (diameter of the photoresist array) determines the crystal size. Four keys are guaranteed to achieve the controllable growth of perovskite nanocrystals, including (1) process stability to guarantee uniform photoresist patterning over a large area for accurate crystal positioning; (2) substrate engineering to ensure uniform crystallization such as flatness of the substrate and wettability at different locations; (3) matching of coating speed and evaporation speed (substrate temperature); (4) keep the gap between the blade and the substrate consistent at different positions. The slight inconsistency is due to the spontaneity and randomness of crystallization. Figure 3d shows the histogram of perovskite nanoplate sizes on the three samples described in Figure 3a–c, in which 100 crystals are analyzed for each sample. The average crystal sizes (measured by diagonal length) are 94.8, 150, and 247 nm, with a size variation coefficient of 8.7%, 5.9%, and 9.7%, respectively. If measured by the side length of the crystal, the crystal sizes of these perovskites are as small as 67, 106, and 174 nm, respectively. These results not only demonstrate the feasibility of the strategy to precisely control perovskite crystal size at the nanometer scale by changing the size of the photoresist pattern but also illustrate the uniform morphology of the perovskite single crystal arrays. Figure 3e–g shows the EDS mapping of the above samples, showing that Cs, Pb, and halide elements are uniformly distributed in the crystal. Taking CsPbBr_3_ as an example, the proportions of each element are shown in Table S3 (Supporting Information), demonstrating the chemical composition of perovskite crystals. Cs, Pb, and Br are evenly distributed in the perovskite crystal, showing a ratio of 1:0.997:2.668, which is close to the ideal stoichiometry of 1:1:3. The ability to generate perovskite single crystal arrays with controllable size at the nanometer scale and different compositions offers an opportunity for the nanodevices such as advanced high‐pixel‐density display and sensing chips.

**Figure 4:**
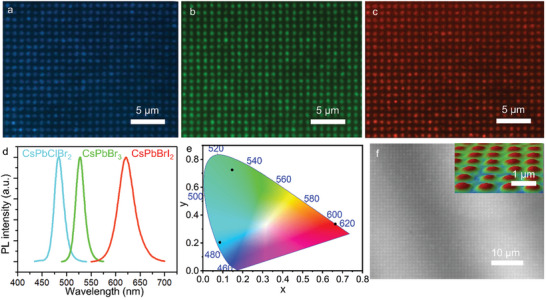
Fluorescence images of the as‐synthesized a) CsPbClBr_2_, b) CsPbBr_3,_ and c) CsPbBrI_2_ single crystal arrays. d) PL spectra of CsPbClBr_2_ (blue), CsPbBr_3_ (green), and CsPbBrI_2_ (red) single crystal arrays. e) CIE color coordinates corresponding to multi‐color perovskite single crystal arrays. f) Microscopy image of CsPbBr_3_ single crystal array. The insert image demonstrates the 3D profile of single crystals.

A series of optical characterizations are performed to confirm the optical properties of multi‐color perovskite single crystal arrays. Perovskite nanocrystal arrays emitting different colors can be achieved by changing the ratio of halide elements. **Figure** **4**a–c demonstrates the fluorescence images of the CsPbClBr_2_, CsPbBr_3,_ and CsPbBrI_2_ single crystal arrays under the excitation of 365 nm UV light, emitting blue, green, and orange light, respectively. The luminescence color shifts to blue as the chlorine ratio increases and to red as the iodine ratio rises. The PL spectra of perovskite crystal arrays including blue emission (485 nm) from CsPbClBr_2_, green emission (521 nm) from CsPbBr_3,_ and orange emission (629 nm) from CsPbBrI_2_ are shown in Figure 4d. As shown in Figure 4e, the international commission on Illumination (CIE) chromaticity figure shows color coordinates of multi‐color perovskite crystal arrays, which are labeled as black dots. Figure 4f demonstrates the microscopy image of CsPbBr_3_ single crystal array using the white light interferometers, and the 3D profile of single crystals is shown in the insert image and and Figure S10 (Supporting Information). In addition to inorganic perovskites, this strategy can also be extended to organic‐inorganic hybrid perovskite materials, and MAPbBr_3_ and FAPbBr_3_ single crystal arrays can also be prepared, as shown in the Figure S11 (Supporting Information).

**Figure 5:**
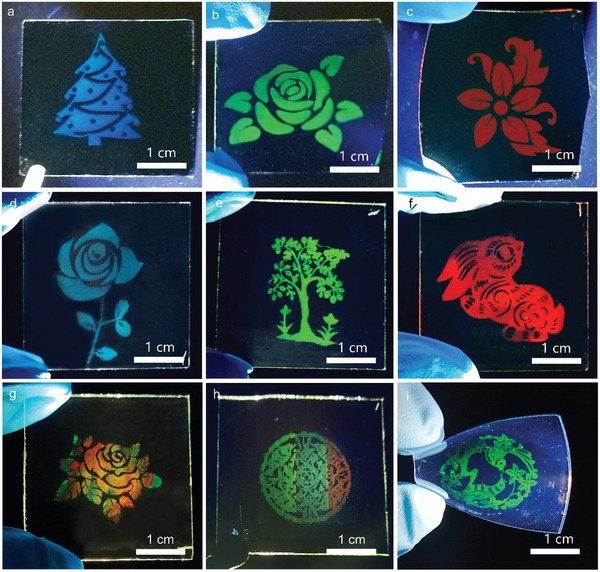
Typical fluorescent images of the centimeter‐level complex perovskite patterns on SiO_2_ substrate with a) blue, b) green, and c) red emission under 365 nm UV excitation. d‐f) PL images of intricate perovskite patterns on glass substrates with different emissions. The fluorescent images of multi‐color patterns with g) random and h) controllable components. i) The fluorescent image of the pattern on a flexible substrate transferred by thermal nanoimprinting.

Finally, complex perovskite nano/micro‐patterns are fabricated to further validate the potential in device applications. Periodic nanopillar arrays are first prepared by interference lithography, and then a secondary exposure using projection lithography defines the image pattern at a larger scale. After patterning the wettability on the substrate surface using two FDTS treatments with the photoresist pattern as a mask, the perovskite solution is blade‐coated to form the perovskite nanocrystal arrays exhibiting the projected image. This strategy is compatible with a variety of substrates, such as glass substrates and Si substrates (Figure S12, Supporting Information). **Figure** **5**a–c shows uniform and bright complex perovskite patterns on SiO_2_ substrates, such as Christmas tree, rose, peach blossom, which are excited under 365 nm UV light. As shown in Figure 5d–f, multi‐color perovskite patterns including rose, tree and rabbit are successfully realized on a 3^*^3 cm^2^ transparent glass substrate. Moreover, multi‐color fluorescent patterns with random or controllable components are demonstrated in Figure 5g,h. The preparation of multi‐color patterns is similar to that of single color. The only difference is that various inks are blade coated on the wettability‐contrast substrate with nano‐photoresist patterns. For Figure 5g, we randomly drop different color solutions into the gap and substrate, then the solutions merge and diffuse during the coating process, resulting in random components and color emissions at different positions of the pattern. To construct a pattern with controllable components (Figure 5h), three gradient component solutions are injected into the gap, and there is diffusion and fusion only at the junction of the two adjacent solutions, thus the pattern with gradient color emission is realized. More importantly, the method can also be extended to flexible substrates. By thermal imprinting, the perovskite pattern is completely transferred to flexible COC substrate by adjusting imprinting temperature and time, as shown in Figure 5i. Proper imprinting temperature is essential as too high temperature will damage the transferred perovskite crystals while too low temperature will result in incomplete transfer and low yield. The transfer pressure also plays a vital role in achieving a high‐yield transfer while minimizing the risk of causing damage to the substrate or the nanocrystals. The rigid donor substrate where the perovskite nanocrystals were initially formed and the flexible acceptor substrate onto which the nanocrystals were transferred, as shown in Figure S13 (Supporting Information). Almost no residue perovskite crystals were found left on the rigid substrate, and complex patterns were completely transferred to the flexible substrate. In summary, the one‐step strategy to fabricate nanoscale perovskite full‐color single crystal arrays, holds the advantages of rapidity, scalability, precision, and compatibility (Table S4, Supporting Information), showing great potential in optoelectronic devices and other application scenarios.

## 3 Conclusion

This work employs well‐developed interference lithography and solution‐processed blade coating to achieve stable, fast, and large‐area patterning of perovskite single‐crystal arrays at the nanometer scale. By optimizing the wettability contrast pattern on the substrate surface and the size of the photoresist pattern, remarkably high‐resolution and size‐controllable (90–260 nm) perovskite single crystal arrays can be directly realized and in situ crystallized in the ambient environment over wafer‐scale area. This strategy can be widely extended to various types of halide perovskites of different compositions, which is proved by the uniform, bright, and colorful complex patterns that show excellent photoluminescence performance. In addition, various rigid substrates including SiO_2_, Si, glass, and flexible substrates such as COC are compatible with this strategy. This work provides a reliable method to fabricate high‐resolution, high‐crystalline‐quality, and large‐scale perovskite arrays in a high‐throughput manner, laying a solid foundation for future perovskite‐based nano‐optoelectronic devices and flexible full‐color displays.

## 4 Experimental Section

### Materials

Cesium bromide (CsBr, 99.9%), Cesium chloride (CsCl, 99.9%), Cesium iodide (CsI, 99.9%), Methylammonium Bromide (MABr, 99.9%), Formamidinium Bromide (FABr, 99.9%), Lead chloride (PbCl_2_, 99.9%), lead bromide (PbBr_2_, 99.9%), lead iodide (PbI_2_, 99.9%) and 2‐Phenylethanamine bromide (PEABr, 99%) were purchased from Xi'an polymer light technology corporation. 18‐Crown‐6, dimethyl sulfoxide (DMSO), Dimethylformamide (DMF) and cyclohexyl‐2‐pyrrolydone (CHP) were purchased from Aladdin Reagent. Trichloro(1H,1H,2H,2H‐perfluorooctyl)silane (FDTS) was purchased from Sigma–Aldrich. All reagents were used without further purification.

### The Preparation of Multi‐Color Perovskite Inks

0.2 m CsPbClBr_2_ precursor (blue) was fabricated by mixing 0.2 mmol PbBr_2_, 0.2 mmol CsCl,0.08 mmol PEABr and 3.5 mg Crown in 1 mL DMSO at 80 °C hot plate for 3 h with constant stirring in the glove box. Similarly, mix 0.2 mmol PbBr_2_, 0.2 mmol CsBr, 0.08 mmol PEABr and 3.5 mg Crown in 1 mL DMSO to prepare 0.2 m CsPbBr_3_ precursor (green), and 0.2 mmol PbBr_2_, 0.2 mmol CsBr, 0.08 mmol PEABr and 3.5 mg Crown were mixed in 1 mL DMSO to fabricate 0.2 M CsPbBrI_2_ precursor (red), then stir the two precursors under the same conditions. Equimolar amount of MABr/FABr and PbBr_2_ were dissolved in a mixed solvent of DMF and CHP with a volume ratio of 9:1. After stirring on a hot plate at 60 °C for 3 hours, 0.5 mol L^−1^ MAPbBr_3_ and FAPbBr_3_ precursors can be obtained.

### Fabrication of Patterned Wettability‐Contrast Substrates

The Si/SiO_2_ substrate was sequentially cleaned with acetone, isopropyl alcohol, ethanol, and deionized water for 8 min each in an ultrasonic cleaner and then dried with nitrogen. AZ 701 photoresist was spin coated on the clean Si/SiO_2_ substrate at a rotation speed of 3000 r s^−1^ for 1 min, and then heated on a 90 °C hot plate for 1 min. Then the interference lithography was introduced to achieve an arrayed photoresist pattern, and then heat the substrate at 110 °C for 2 min. After developing in AZ726 developer for 1 min, the patterned substrate was then placed in a FDTS atmosphere at 100 °C for 15 min to assemble a lyophobic layer on the areas not covered by the photoresists. Subsequently, clean the above substrate with acetone, IPA, alcohol, and water in sequence. The second lyophobic treatment was performed on the substrate at 25 °C for 2 min so that the lyophilic area covered by the photoresist became slightly lyophobic to form a substrate with appropriate wettability differences.

### The Synthesis of Size‐Controlled Perovskite Single‐Crystal Arrays

First, the wettability‐contrast substrate was placed on a hot plate at 35 °C, and then 5 µl of perovskite precursor was dropped on the surface of the substrate. The solution was separated into many tiny droplets by the capillary force of the blade at a speed of 625 µm s^−1^, which were confined to the lyophilic regions. Afterward, anneal the substrate on a hot plate at 100 °C for 1 min. The size of the perovskite crystal can be further adjusted by regulating the size of the photoresist pattern. The size of perovskite crystal increases with the rise of the size of the photoresist pattern.

### Thermal Imprinting Transfer of Perovskite Pattern

A piece of FDTS‐treated glass was used as a bottom substrate, which not only ensures the flatness of the flexible substrate after imprinting but also facilitates the peeling due to its hydrophobicity. Then a blank COC, the sample with the perovskite pattern facing down, a piece of polytetrafluoroethylene (PTFE) film as well and a silicone pad were stacked sequentially and placed between the upper and lower platens of the imprinter. After that, the temperature of the platen was lowered to room temperature by a water cooler. Finally, the sample can be taken out and each layer can be easily separated to realize the transfer process of the perovskite pattern from rigid to flexible substrates.

### Characterization

The contact angle measurement was performed using dynamic contact angle measuring devices (DataPhysics Instruments). Photoluminescence Spectrometer (Edinburgh, FLS1000) was applied to study the PL spectra using excitation of 405 nm UV light. Absorption spectra was collected by UV–VIS‐NIR spectrometer (Agilent Cary 5000). The surfaces and structures of single crystals were characterized by field emission scanning electron microscopy (Hitachi, S‐4800). As for PL images, the fluorescent microscope (LEICA DFC7000T) was utilized to capture them. The thickness profile of a single perovskite crystal was obtained by Bruker Atomic Force Microscope (MultiMode 8). The X‐ray diffraction (XRD) system (Rigaku, SmartLab, 9kW X‐ray diffactometer) was used to examine crystalline structures. Besides, the large‐area 3D profiles of perovskite single‐crystal arrays were measured by Bruker white light interferometer.

## Conflict of Interest

The authors declare no conflict of interest.

## Supporting information



Supporting Information

## Data Availability

The data that support the findings of this study are available from the corresponding author upon reasonable request.
