# Supplementary material for: One‐step Centimeter‐Scale Growth of Sub‐100‐nm Perovskite Single‐Crystal Arrays in Ambient Air for Color Painting
Source: Adv Sci (Weinh). 2025 Feb 3;12(12):2415105. doi: 10.1002/advs.202415105 (PMC11948079; doi:10.1002/advs.202415105)
Supplement: Supplementary file 1 — Supporting Information [file ADVS-12-2415105-s001.docx]

Supporting Information

One-step Centimeter-scale Growth of Sub-100-nm Perovskite Single-Crystal Arrays in Ambient Air for Color Painting

Guannan Zhang, Zhao Sun, Zhuofei Gan, Chuwei Liang, Liyang Chen and Wen-Di Li *


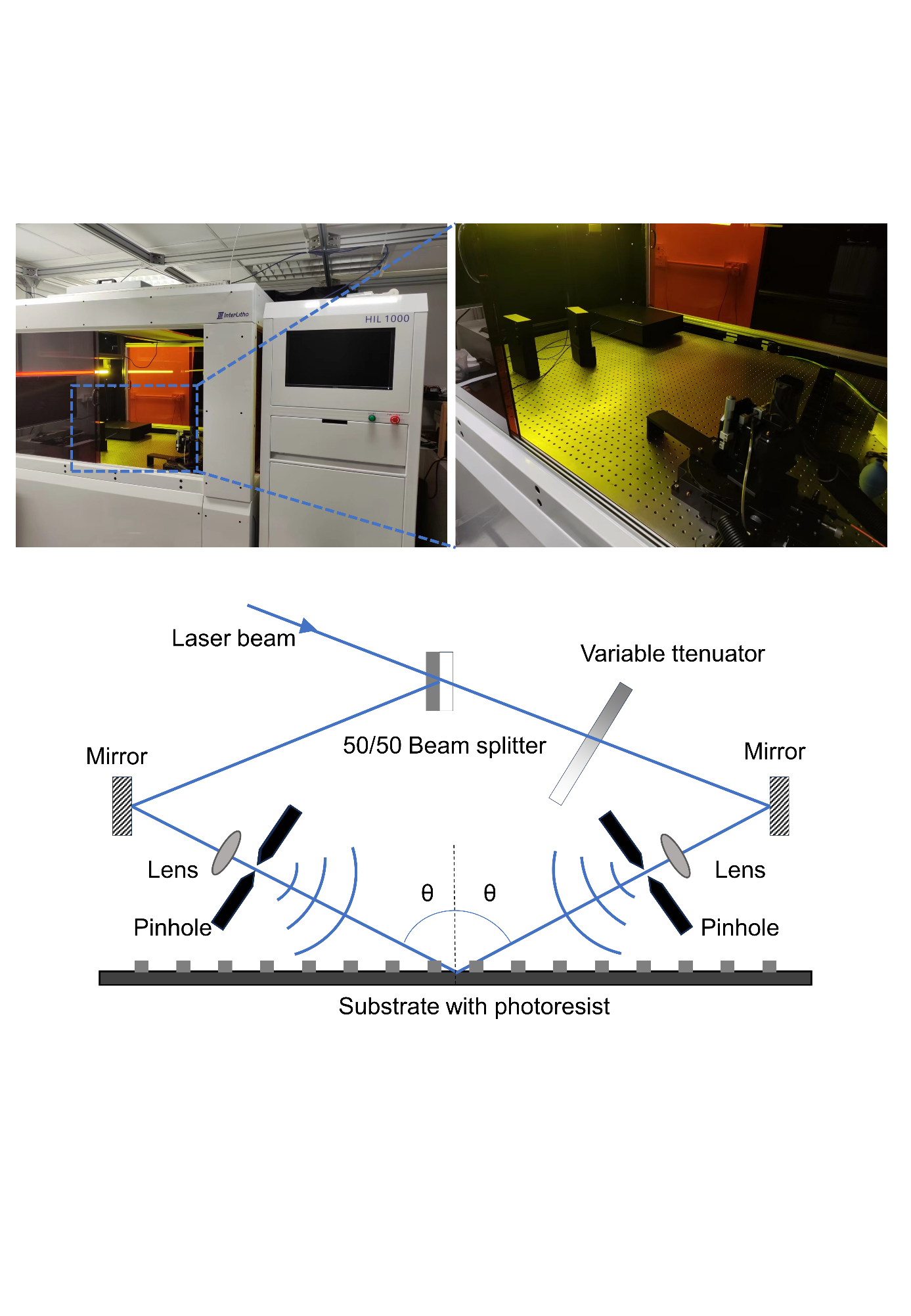


**Figure S1.** Physical pictures of interference lithography equipment.


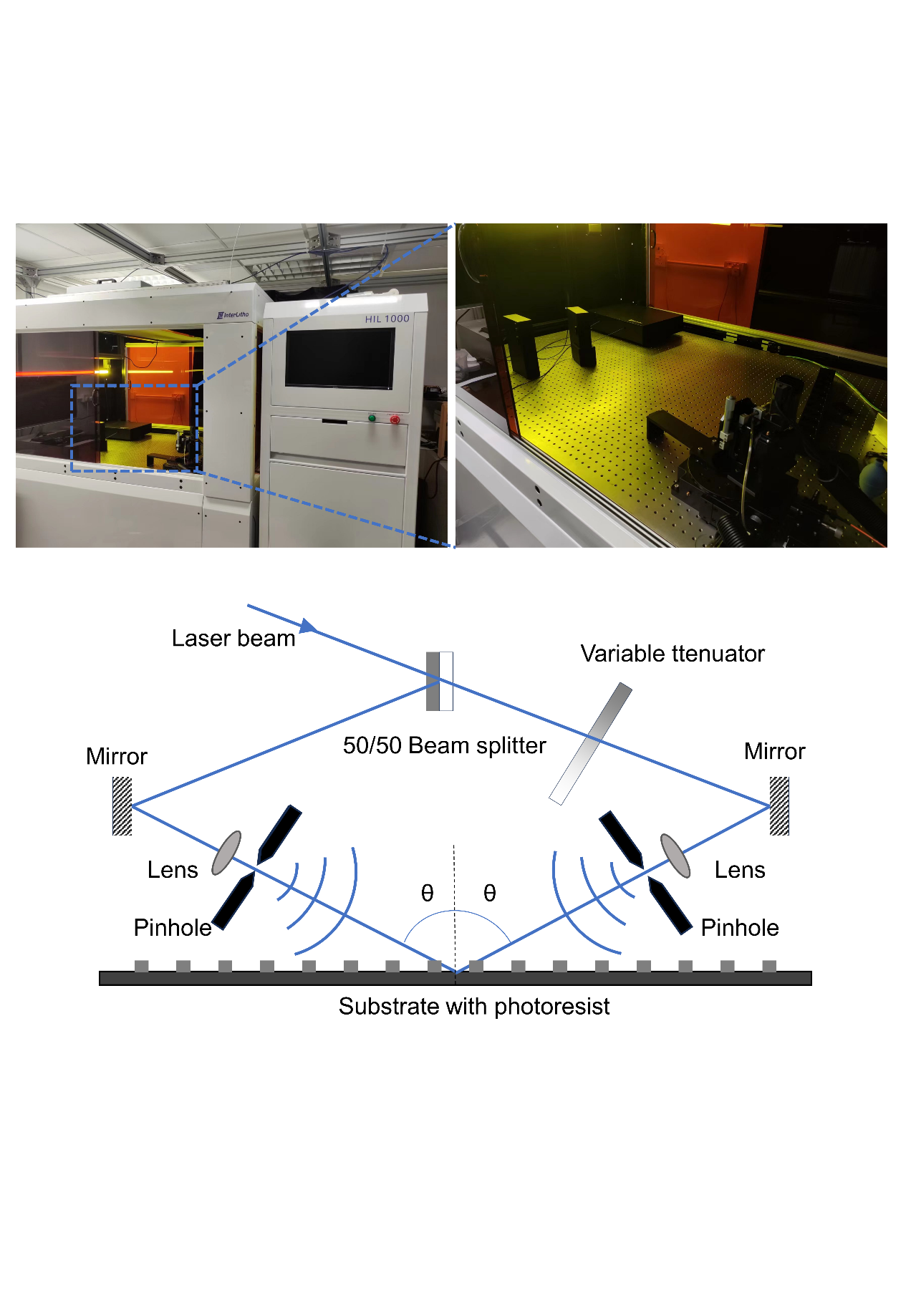


**Figure S2.** Principle image of interference lithography.

**Supplementary Note 1. Principle of interference lithography.**

As shown in **Figure S2**, the laser beam is divided into two beams with equal intensity by a 50/50 laser beam splitter. The two light waves superimpose on the substrate and interfere with each other to form an interference pattern, which is recorded in the photoresist layer. After development, a photoresist pattern with periodic lines or hole/dot arrays is formed.


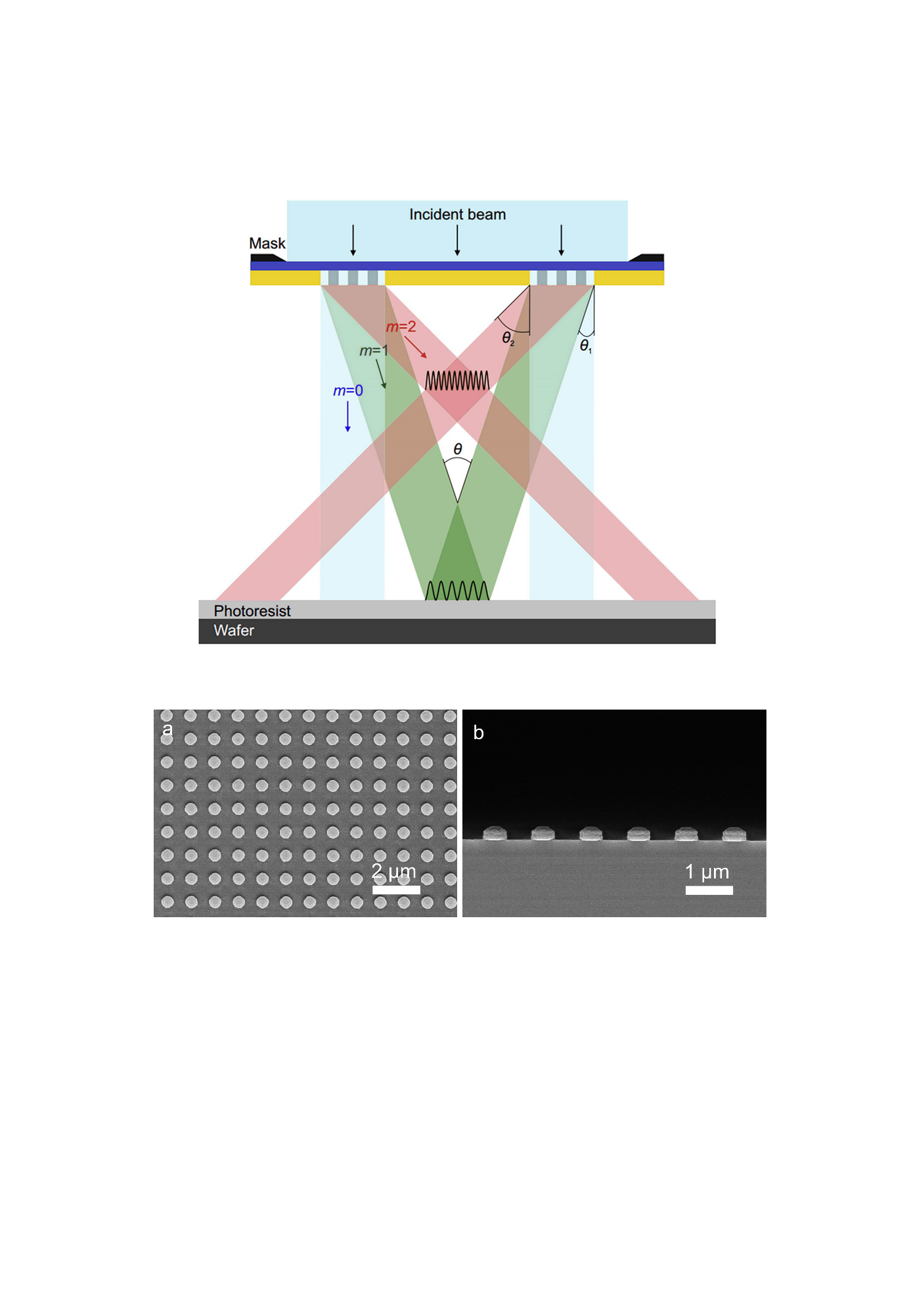


**Figure S3.** a) Top-view SEM images and b) side-view SEM images of two-dimensional nanopillar arrays.


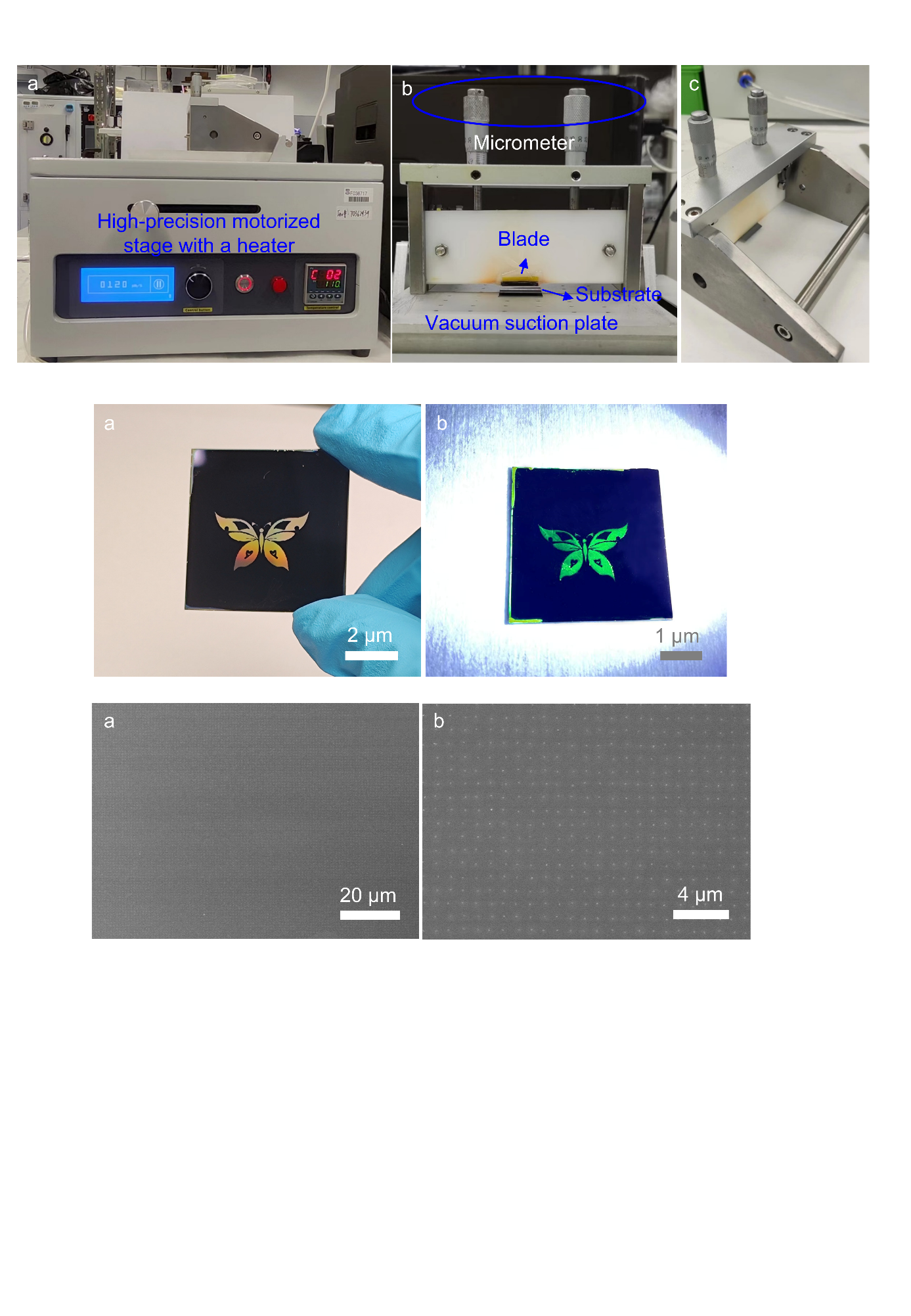


**Figure S4.** Physical pictures of blade coating system. a) High-precision motorized stage with a heater. b-c) details about the blade.

**Supplementary Note 2. Blade-coating system.**

The blade-coating system consists of a blade, hot plate, and control system as well as other auxiliary equipment, as shown in Figure S4. The functions of blade and hot plate are the deposition of thin-ﬁlm and the evaporation of solvent, respectively. The control system mainly controls the height between blade and substrate, the moving speed of blade, and the temperature of hot plate. When the blade-coating equipment works, the precursor solution drops onto the substrate, and then the blade with desired speed moves from one end of the substrate to the other end to complete the preparation of perovskite ﬁlms. Here, we use the anti-stick treated Si sheet as the blade, which is fixed on the custom bracket with UV glue, and two micrometers can help to precisely adjust the distance between the blade and the substrate. In addition, the temperature range of the substrate is 20 ℃ to 120 ℃, which heats and evaporates the wet perovskite ﬁlm in time.


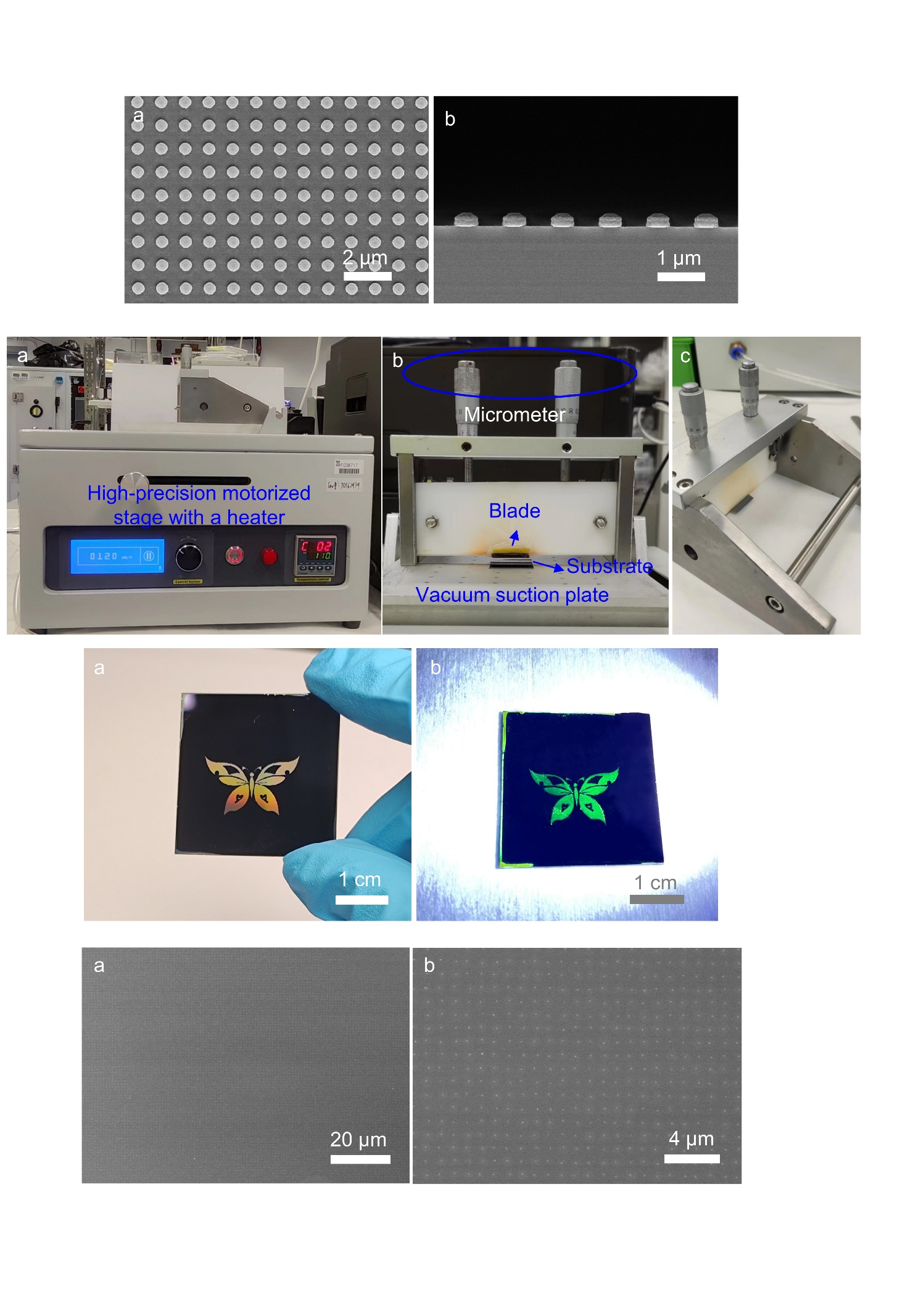


**Figure S5**. a) Physical picture of ‘butterfly’ photoresist pattern. b) Physical picture of the sample after blade coating.


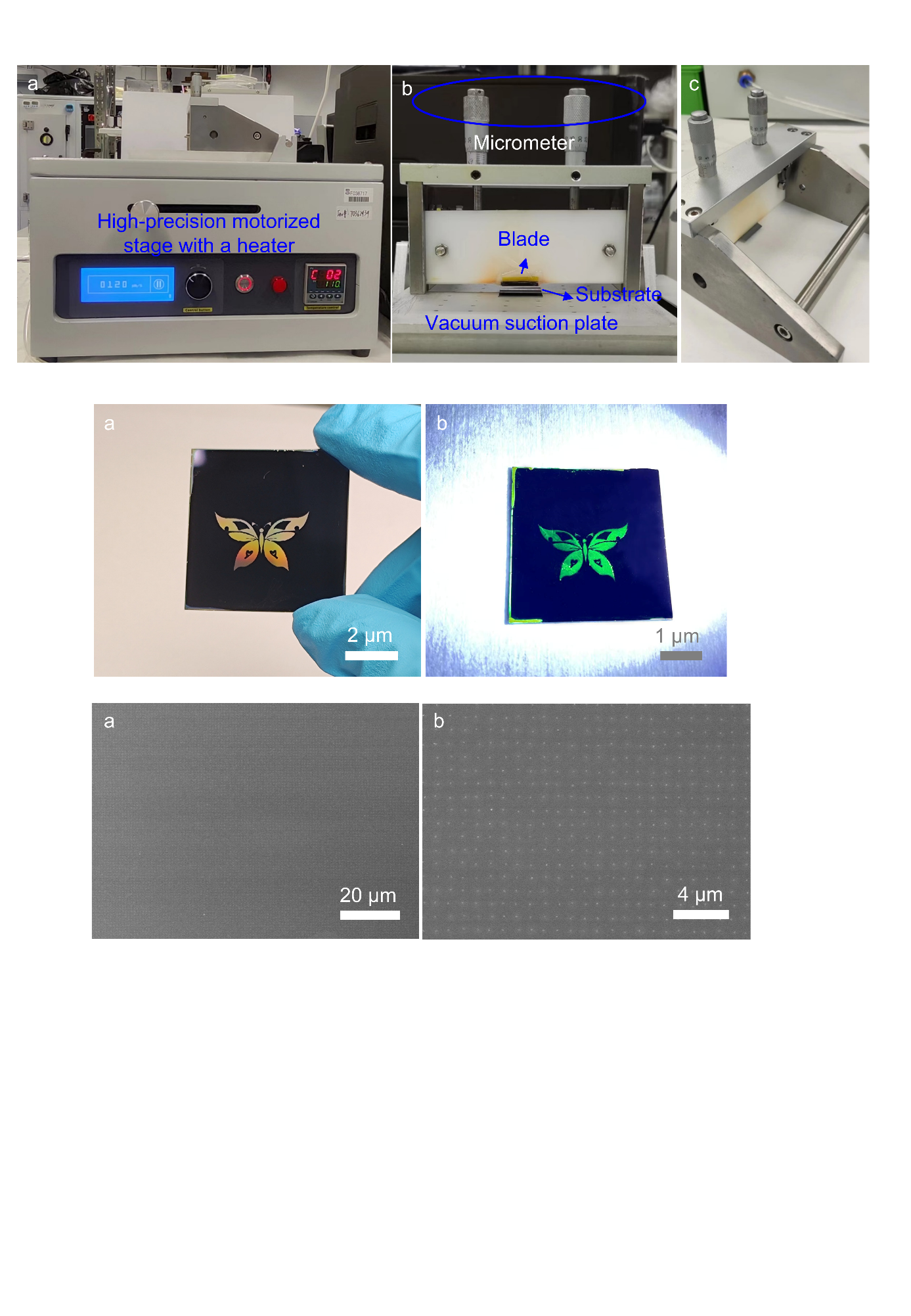


**Figure S6.** The SEM images of perovskite single crystal array in the butterfly pattern.

**Supplementary Note 3. Evaporation process of solution on wettability-contrast substrate.**

During the process of solvent evaporation, the outward convective velocity *v* can be described as the simplified Navier-Stokes equation.^[1]^ (Equation S1)

$$\begin{aligned} v=\frac{1}{\rho lh}\int_{0}^{l} J_{e}\left( h \right)ldl\#\left( S1 \right) \end{aligned}$$

Where $\rho$ is the density of solution, $l$ represents the horizontal distance from the bulk ink, and *h* is the meniscus height. In addition, $J_{e}$ describes the evaporative flux of solvent, which can be represented as the nonequilibrium one-sided model (Equation S2).

$$\begin{aligned} J_{e}\left( h \right)=\frac{1}{h+K+W}\#\left( S2 \right) \end{aligned}$$

Where *K* is nonequilibrium parameter, and *W* is the thermal effect, representing by the Equation S3.

$$\begin{aligned} W=\frac{k_{l}t_{s}}{k_{s}t_{l}}\#\left( S3 \right) \end{aligned}$$

Where $k_{l}$ and $k_{s}$ demonstrate the thermal conductivity of liquid and substrate, respectively. And $t_{l}$ and $t_{s}$ are the liquid thickness and substrate thickness.

According to above equations, $v$ at the edge of meniscus increases precipitously, which means that the solvent evaporates quickly on lyophilic substrates while the evaporation rate is rather slow on lyophobic treated substrates, resulting in the selective growth of perovskite single crystals in relatively lyophilic regions.


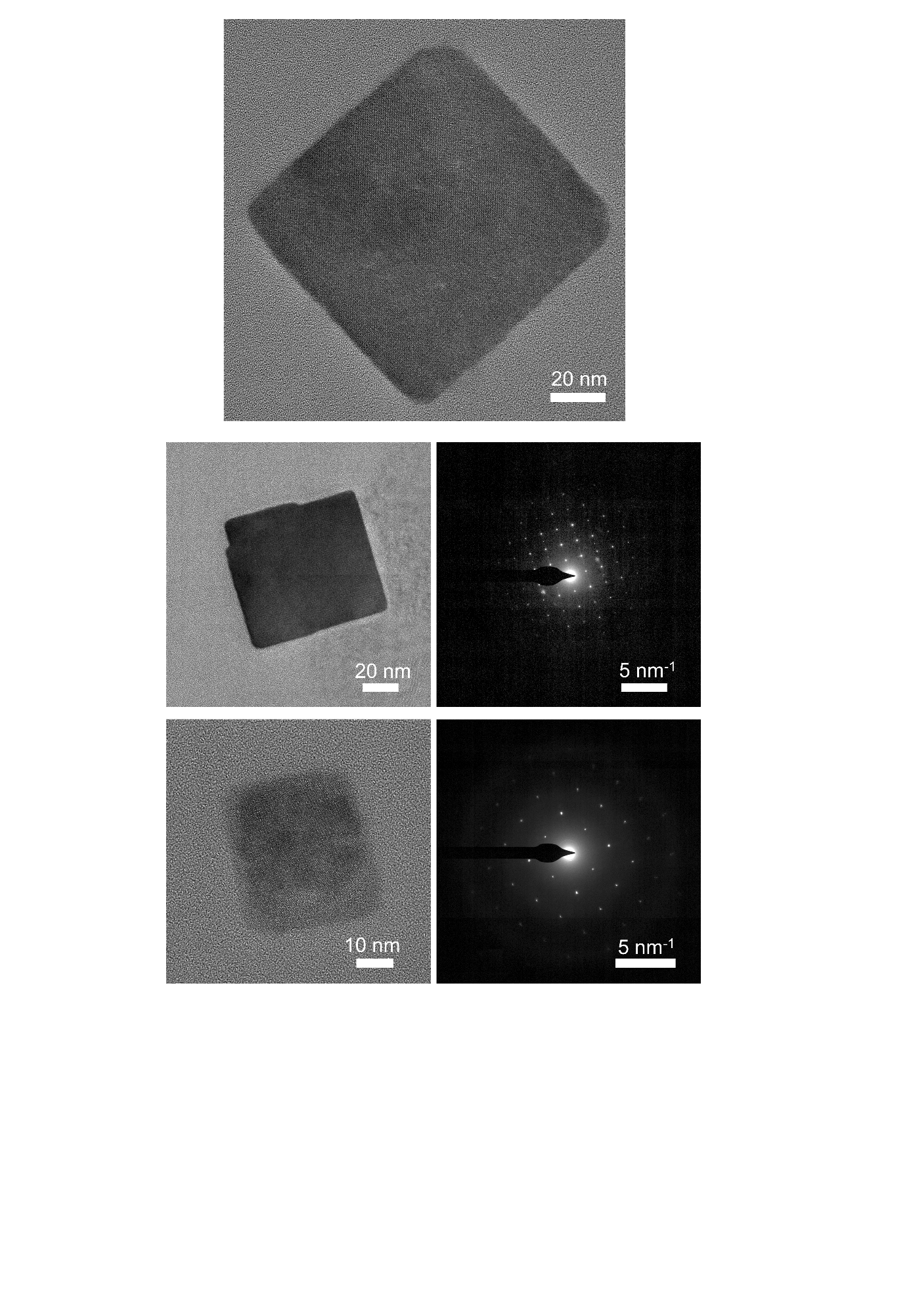


**Figure S7**. The high-resolution TEM (HRTEM) image of a single CsPbBr_3_ crystal.


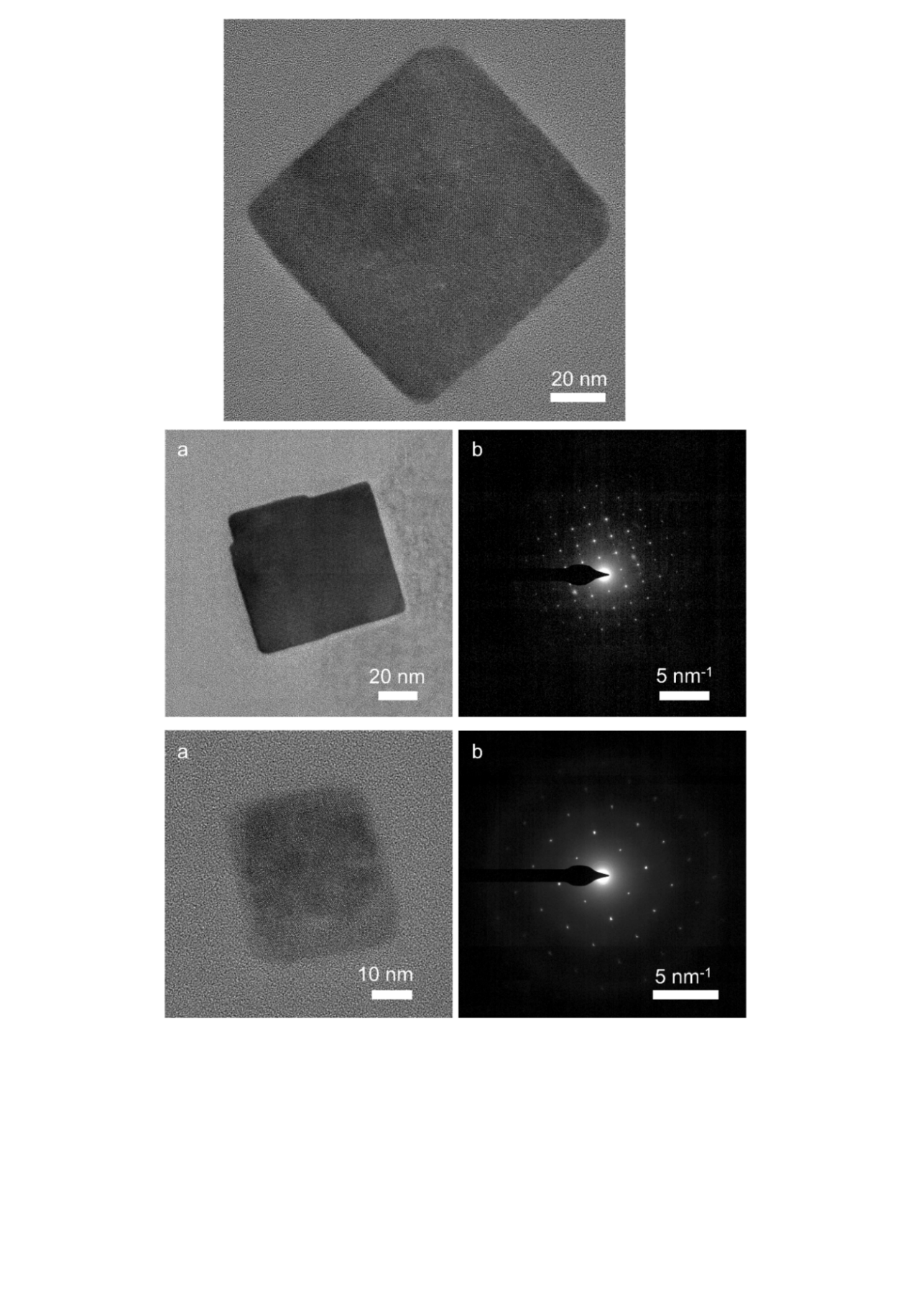


**Figure S8**. a) The high-resolution TEM (HRTEM) image and b) selected area electron diffraction (SAED) pattern of a single CsPbClBr_2_ crystal.


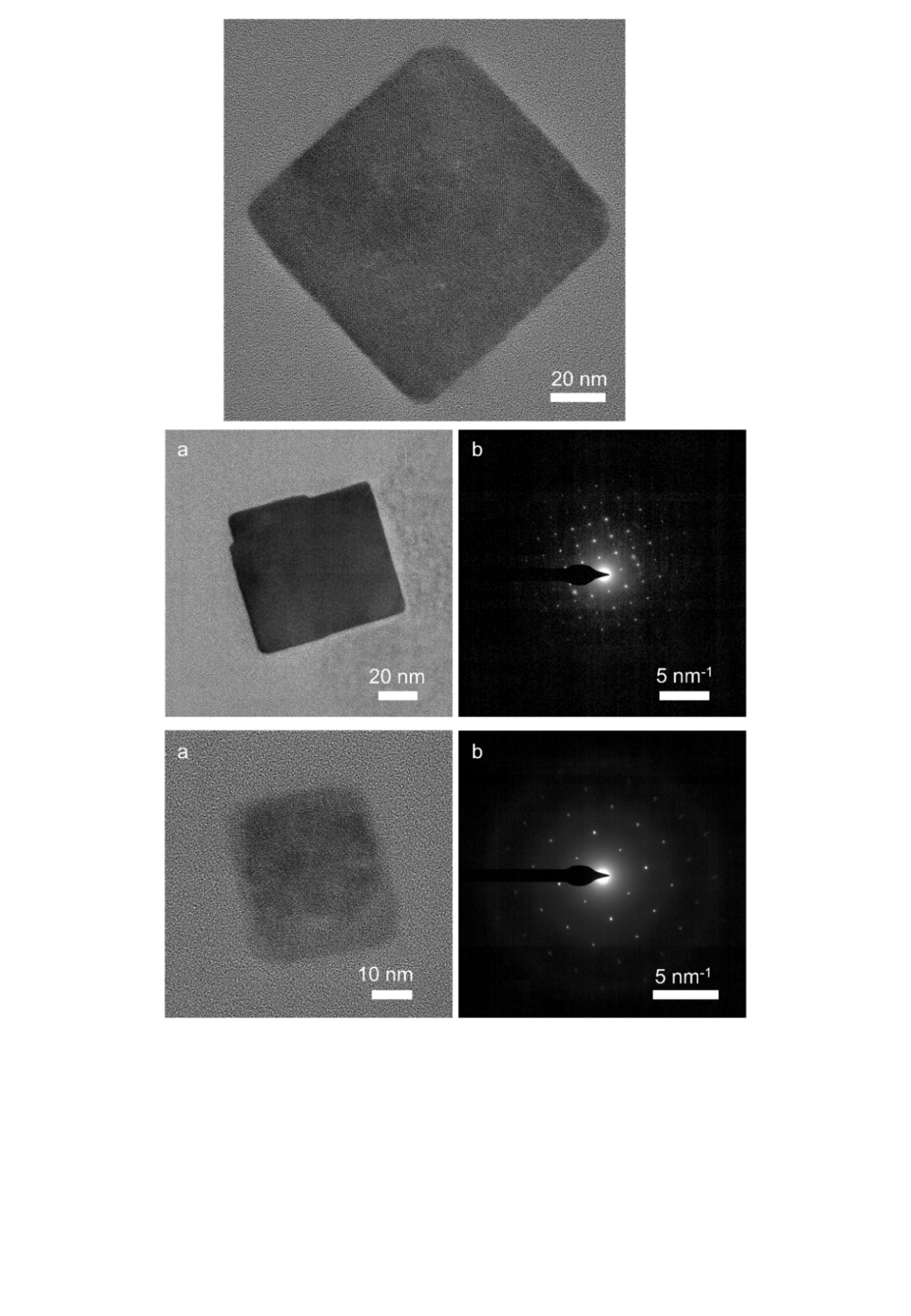


**Figure S9.** a) The high-resolution TEM (HRTEM) image and b) selected area electron diffraction (SAED) pattern of a single CsPbBrI_2_ crystal.

**
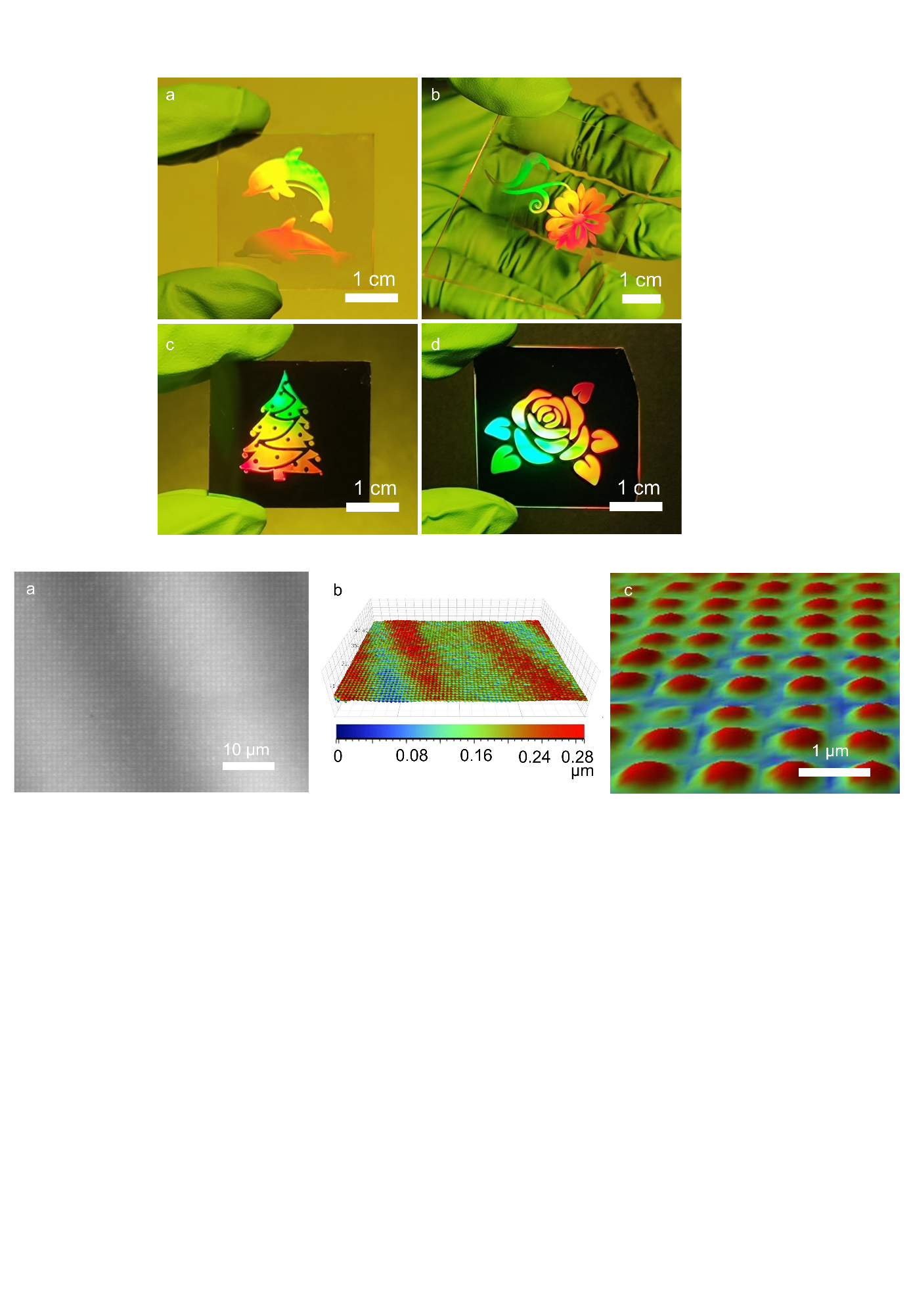
**

**Figure S10.** 3D contour image of CsPbBr_3_ single crystal array.


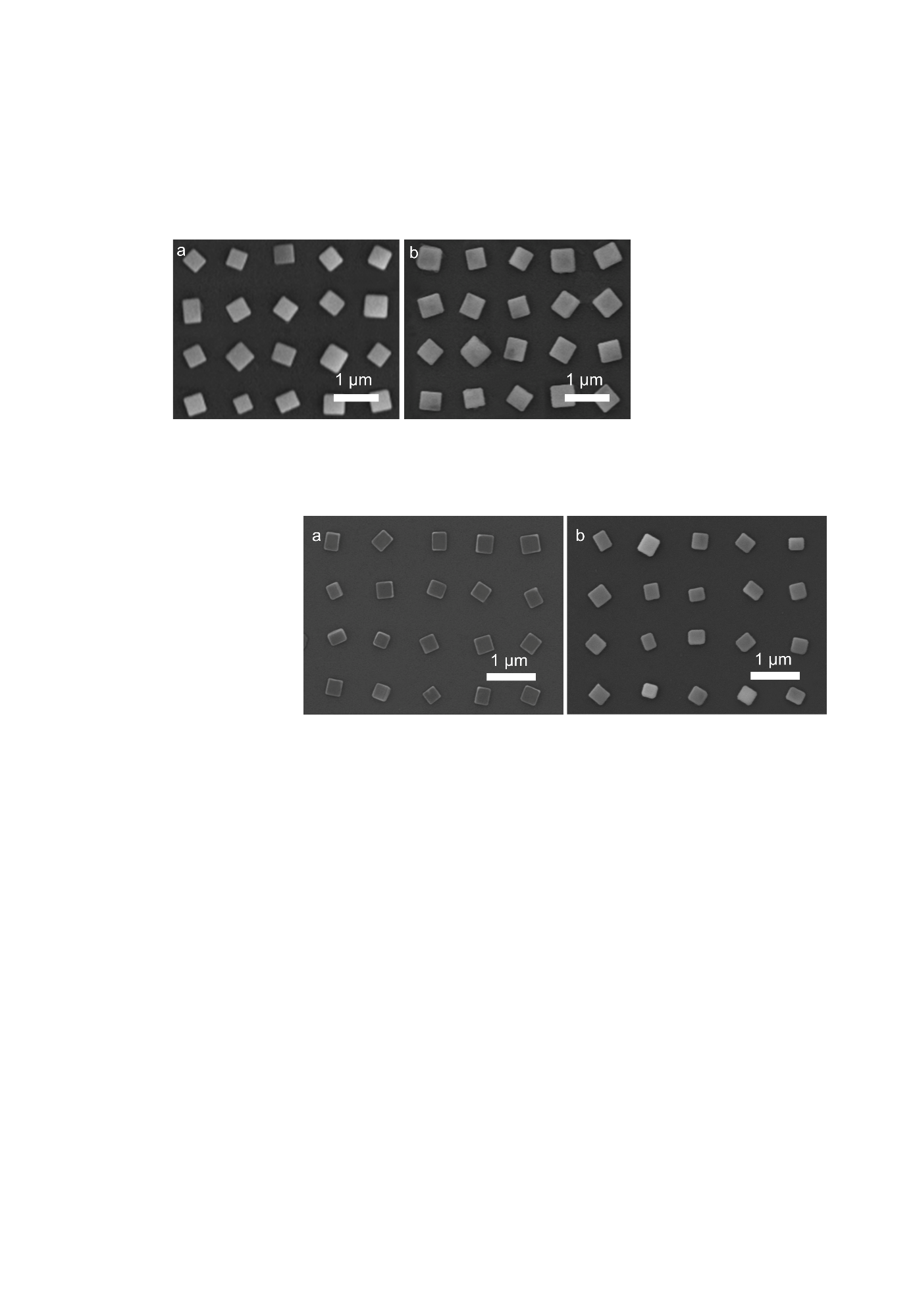


**Figure S11.** SEM images of a) MAPbBr_3_, b) FAPbBr_3_ single crystal arrays.

**
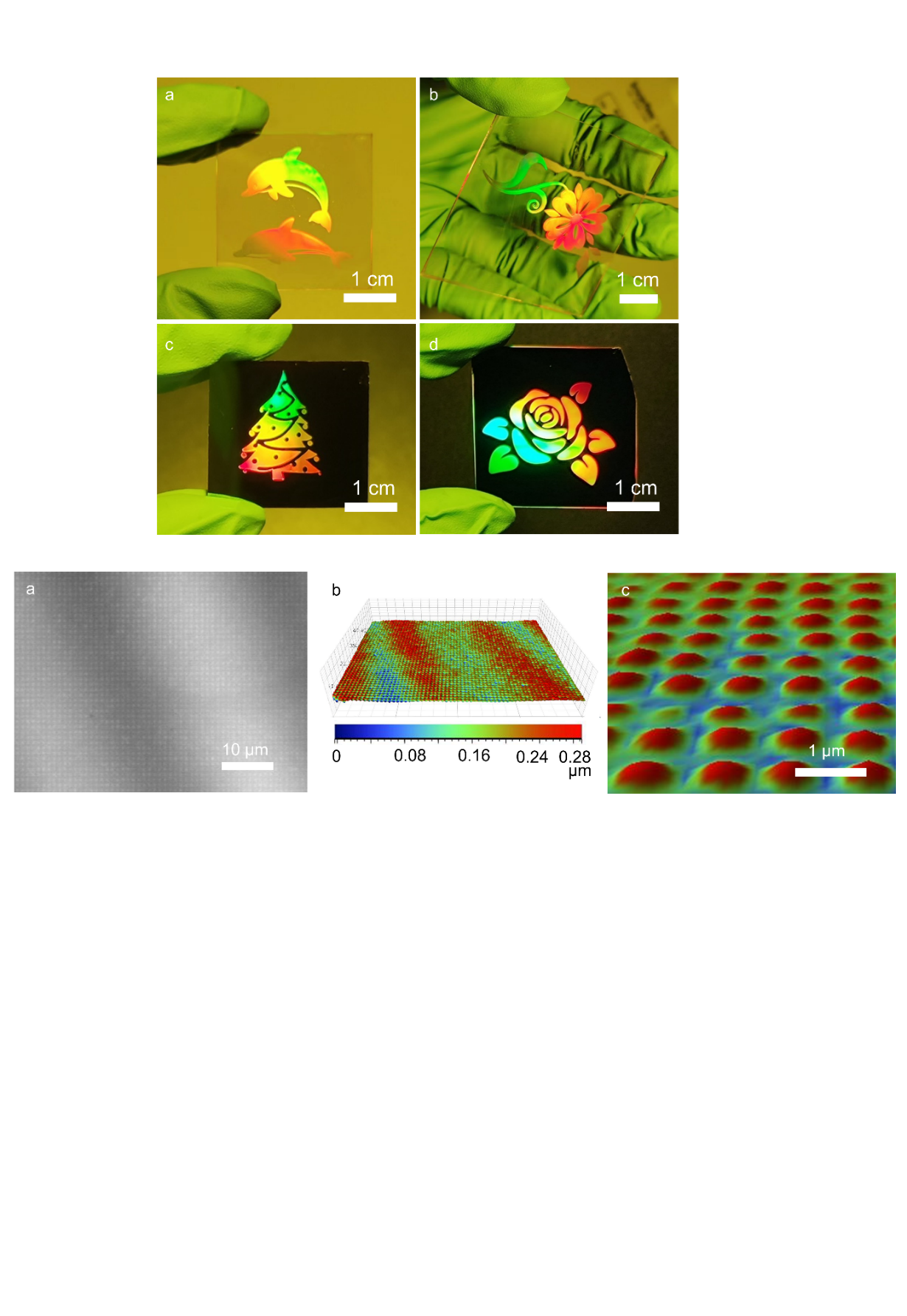
**

**Figure S12.** a-b) Complex photoresist patterns on glass substrates. c-d) Complex photoresist patterns on SiO_2_ substrate.

**
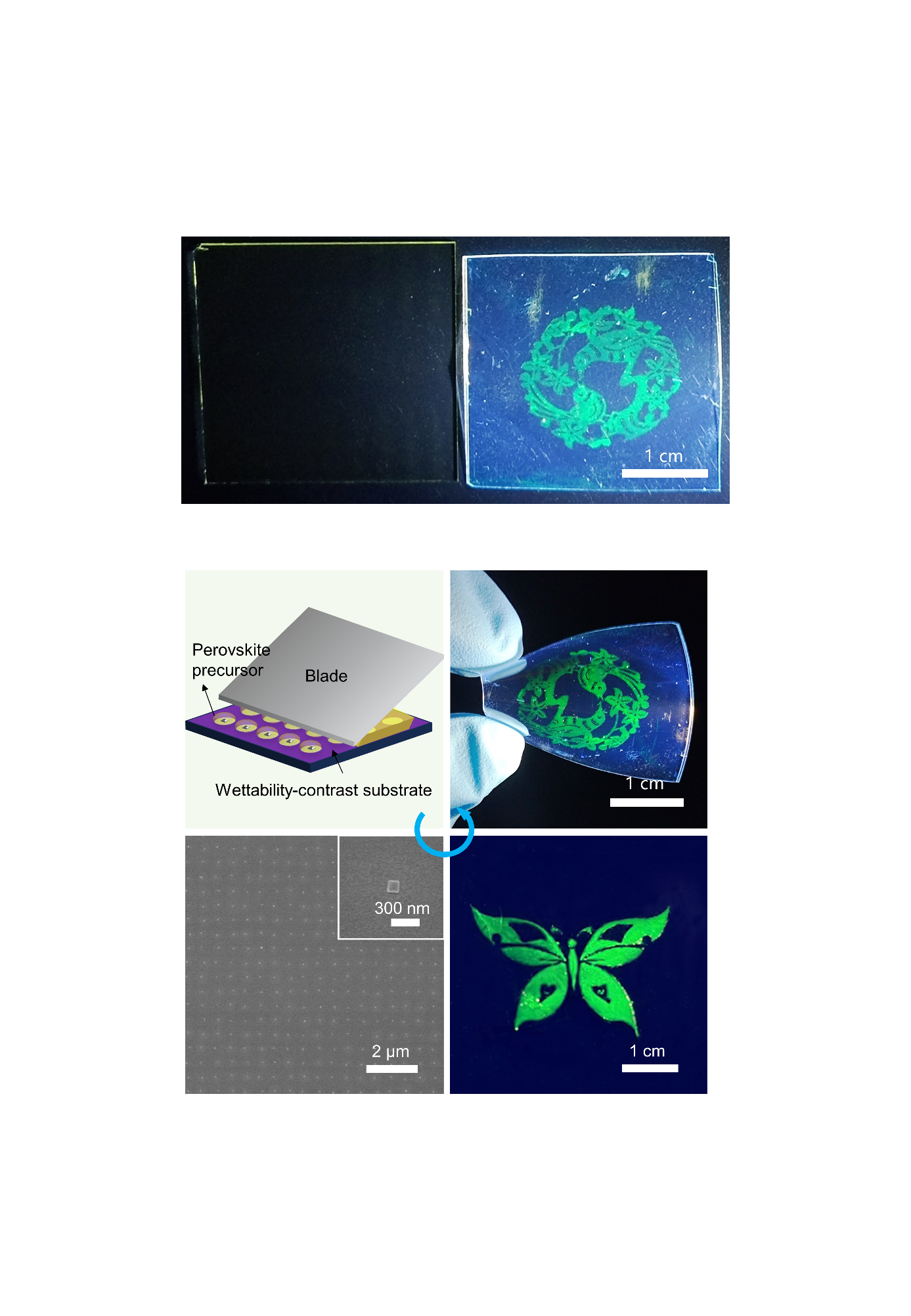
**

**Figure S13.** The fluorescent pattern of ‘magpie’ on rigid and flexible substrates after thermal imprinting transfer.

**Table S1.** The effects of fluorination time on the contact angle of the substrate at 25 ℃.

| Fluorination time (s) | 0 | 10 | 15 | 30 | 60 | 120 | 180 | 240 | 360 | 480 | 600 | 900 |
| --- | --- | --- | --- | --- | --- | --- | --- | --- | --- | --- | --- | --- |
| Contact angle (°) | 0.5 | 32.5 | 37 | 52 | 54 | 57 | 63 | 68 | 71 | 75 | 78 | 81 |

**Table S2.** The effects of fluorination time on the contact angle of the substrate at 100 ℃.

| Fluorination time (s) | 0 | 5 | 10 | 15 | 30 | 60 | 120 | 240 | 360 | 480 | 600 | 900 |
| --- | --- | --- | --- | --- | --- | --- | --- | --- | --- | --- | --- | --- |
| Contact angle (°) | 0.8 | 58 | 70 | 80 | 81 | 81.5 | 83 | 83.4 | 85 | 87.5 | 88 | 89 |

**Table S3.** TEM-EDS analysis of a single CsPbBr_3_ crystal.

|  | Element | Family | Atomic fraction | Atomic error | Mass fraction | Mass error |
| --- | --- | --- | --- | --- | --- | --- |
|  | - | - | % | % | % | % |
|  | Br | K | 57.77 | 2.41 | 39.26 | 2.39 |
|  | Cs | L | 21.65 | 2.06 | 24.47 | 2.31 |
|  | Pb | L | 21.58 | 2.00 | 36.27 | 2.86 |

**Table S4.** Representative methods to fabricate perovskite micro/nano-pattern arrays.

| Material | Process | Single/ Mutil-color | Feature size | Single crystal/ polycrystalline | Efficiency | Area | Rigid/flexible | References |
| --- | --- | --- | --- | --- | --- | --- | --- | --- |
| (PEA)_2_PbX_4_ | Blade coating on lithography-based template | Mutil | ~ 5 μm | Single crystal | High | mm-scale | Rigid | [1] |
| MAPbX_3_ | Blade coating and vapor-phase conversion on lithography-based template | Mutil | ~ 5 μm | Single crystal | High | cm-scale | Rigid | [2] |
| CsPbBr_3_ | Spin coating on EBL-based template | Single | <50 nm | Single crystal | Low | μm-scale | Rigid | [3] |
| MAPbBr_3_ | Inkjet printing on liquid PDMS | Single | ~ 3 μm | Single crystal | General | cm-scale | Flexible | [4] |
| MAPbI_3_ | Photolithography and Dry Lift-Off Patterning | Single | ~ 5 μm | Polycrystalline | High | μm-scale | Flexible | [5] |
| MAPbBr_3_ | Spatially confined growth on the as-prepared Au NP-patterned ITO substrate | Single | ~ 10 μm | Single crystal | Low | cm-scale | Rigid | [6] |
| MAPbX_3_ | Wettability-guided screen printing | Single | ~ 20 μm | Polycrystalline | High | cm-scale | Rigid | [7] |
| CsPbX_3_ | Mold-embedded meltgrowth | Mutil | ~1 mm | Single crystal | General | cm-scale | Rigid | [8] |
| MAPbX_3_ | Wettability-guided coating and CVT conversion | Mutil | ~ 200 nm | Single crystal | General | cm-scale | Rigid | [9] |
| CsPbX_3_ | Surface-tension-confined growth | Mutil | ~ 250 nm | Single crystal | High | mm-scale | Rigid | [10] |
| CsPbX_3_ | Blade coating on wettability-contrast template | Mutil | <100 nm | Single crystal | High | cm-scale | Flexible | This work |

Note: X= Cl, Br, I.

**References**

[1] Y. H. Lee, J. Y. Park, P. Niu, H. Yang, D. Sun, L. Huang, J. Mei,L. Dou, *ACS nano* **2023**, 17, 13840.

[2] Z. Xu, X. Han, W. Wu, F. Li, R. Wang, H. Lu, Q. Lu, B. Ge, N. Cheng,X. Li, *Light Sci. Appl.* **2023**, 12, 67.

[3] P. Jastrzebska-Perfect, W. Zhu, M. Saravanapavanantham, Z. Li, S. O. Spector, R. Brenes, P. F. Satterthwaite, R. J. Ram,F. Niroui, *Nat. Commun.* **2023**, 14, 3883.

[4] Z. Gu, Z. Huang, X. Hu, Y. Wang, L. Li, M. Li,Y. Song, *ACS Appl. Mater. Inter.* **2020**, 12, 22157.

[5] B. Xia, M. Tu, B. Pradhan, F. Ceyssens, M. L. Tietze, V. Rubio-Giménez, N. Wauteraerts, Y. Gao, M. Kraft,J. A. Steele, *Adv. Eng. Mater.* **2022**, 24, 2100930.

[6] T. A. Malo, Z. Lu, W. Deng, Y. Sun, C. Wang, A. A. A. Pirzado, J. Jie, X. Zhang,X. Zhang, *Adv. Funct. Mater.* **2022**, 32, 2209563.

[7] K. Wang, Y. Du, J. Liang, J. Zhao, F. F. Xu, X. Liu, C. Zhang, Y. Yan,Y. S. Zhao, *Adv. Mater.* **2020**, 32, 2001999.

[8] C. Li, X. Ye, J. Jiang, Q.Guo, X. Zheng, Q. Lin, C.Ge, S. Wang, J. Chen, Z. Gao, G. Zhang, X. Tao, Y. Liu, *Small* ***2024****, 20, 2401624.*

[9] C.-K. Lin, Q. Zhao, Y. Zhang, S. Cestellos-Blanco, Q. Kong, M. Lai, J. Kang,P. Yang, *ACS nano* **2020**, 14, 3500.

[10] H. Du, K. Wang, L. Zhao, C. Xue, M. Zhang, W. Wen, G. Xing,J. Wu, *ACS Appl. Mater. Inter.* **2019**, 12, 2662.
